# Two-Dimensional Titanium Carbides (Ti_3_C_2_T_x_) Functionalized by Poly(m-phenylenediamine) for Efficient Adsorption and Reduction of Hexavalent Chromium

**DOI:** 10.3390/ijerph17010167

**Published:** 2019-12-25

**Authors:** Linfeng Jin, Liyuan Chai, Weichun Yang, Haiying Wang, Liyuan Zhang

**Affiliations:** 1 School of Metallurgy and Environment, Central South University, Changsha 410083, China; 2 Chinese National Engineering Research Center for Control and Treatment of Heavy Metal Pollution, Changsha 410083, China; 3 Water Pollution Control Technology Key Lab of Hunan Province, Changsha 410004, China; 4 Department of Colloid Chemistry, Max Planck Institute of Colloids and Interfaces, 14476 Potsdam, Germany

**Keywords:** titanium carbides, functionalization, polymerization, hexavalent chromium, adsorption

## Abstract

Titanium carbides (MXenes) are promising multifunctional materials. However, the negative surface charge and layer-by-layer restacking of MXenes severely restrict their application in the field of anionic pollutants, including in hexavalent chromium (Cr(VI)). Herein, Ti_3_C_2_T_x_ MXenes was functionalized through in situ polymerization and intercalation of poly(m-phenylenediamine) (PmPD), then Ti_3_C_2_T_x_/PmPD composites were obtained. Delightedly, Ti_3_C_2_T_x_/PmPD composites exhibited positive surface charge, expanded interlayer spacing, and enhanced hydrophobicity. Furthermore, the specific surface area of Ti_3_C_2_T_x_/PmPD composite was five and 23 times that of Ti_3_C_2_T_x_ and PmPD, respectively. These advantages endowed Ti_3_C_2_T_x_/PmPD composite with an excellent adsorption capacity of Cr(VI) (540.47 mg g^−1^), which was superior to PmPD (384.73 mg g^−1^), Ti_3_C_2_T_x_ MXene (137.45 mg g^−1^), and the reported MXene-based adsorbents. The Cr(VI) removal mechanism mainly involved electrostatic adsorption, reduction, and chelation interaction. This study developed a simple functionalization strategy, which would greatly explore the potential of MXenes in the field of anionic pollutants.

## 1. Introduction

Hexavalent chromium (Cr(VI)) pollution poses a serious crisis to human beings and the ecosystem, due to its high mobility, toxicity, and potential carcinogenicity [1,2]. It is extremely urgent to treat Cr(VI) contamination. In contrast, trivalent chromium (Cr(III)) usually has low levels of toxicity, is immobile, and even is an essential micronutrient for organisms [3,4,5]. At present, adsorption remains an effective method for Cr(VI) remediation [6], which involves the conversion from toxic Cr(VI) to mild Cr(III) in the adsorption process [7,8]. Various adsorbents have been developed, such as biochar [9,10], the metal–organic framework [11,12], nanoscale zero-valent iron [13,14], graphene oxide [15,16], and organic polymer [17,18]. Unfortunately, current adsorbents generally suffer from unsatisfactory removal capacity, a low adsorption rate, and weak reduction capacity. Therefore, the development of novel adsorbents with an outstanding performance is still a paramount challenge.

Transition metal carbides (MXenes) are novel two-dimensional (2D) materials, which were first reported by Yury Gogotsi in 2011 [19,20,21]. Their unique physicochemical properties (such as a layered structure, high hydrophilic surface, and excellent electrical conductivity) endow MXenes with promising advantages in electromagnetic interference shielding [22], energy storage fields [23], and conducting thin films [24]. In recent years, MXenes have received increasing attention in the field of environment owning to their large amounts of surface negative terminations (such as -O, -OH, and -F) [25,26,27]. These negative terminations render MXenes with favorable removal capacity for cationic pollutants. 

However, MXenes still face great challenges in the remediation of anionic pollutants due to the charge repulsion between MXenes and anionic pollutants [28]. Moreover, due to the hydrogen bonding between the surface functional groups, MXenes are always reassembled as tightly as graphene and other 2D materials in practical applications [29,30], which would inevitably decrease the mass transfer efficiency and availability of MXenes [31]. Regulating surface charge and interlayer spacing are feasible approaches to fully explore the potential of MXenes in the field of Cr(VI) remediation. However, there is still no relevant report on this at present.

As a kind of conjugated polymer, poly(m-phenylenediamine) (PmPD) has been widely employed to functionalize matrix materials because of its simple synthesis and abundant amino groups [32,33,34]. In this research, Ti_3_C_2_T_x_ (X = OH, O or F) was selected as the representative of the MXenes family, and the regulation of surface charge and expansion of interlayer spacing of Ti_3_C_2_T_x_ were successfully achieved by in situ polymerization and intercalation of PmPD. Accordingly, a novel MXenes/poly(m-phenylenediamine) (Ti_3_C_2_T_x_/PmPD) composite was obtained and utilized to adsorb Cr(VI) from aqueous solution. Finally, the preparation mechanism and adsorption mechanism were investigated in detail. 

## 2. Material and Methods

### 2.1. Materials

Ti_3_AlC_2_ MAX powder (400 mesh) was purchased from 11 Technology Co., Ltd. m-Phenylenediamine (99.5%) was purchased from Aladdin Reagent. All other reagents were of an analytical grade and were purchased from Sinopharm Chemical Reagent.

### 2.2. Preparation of Ti_3_C_2_T_x_, Ti_3_C_2_T_x_/PmPD, PmPD

Ti_3_C_2_T_x_ was synthesized by etching and delaminating Ti_3_AlC_2_ MAX powder through the typical minimally intensive layer delamination (MILD) approach [20]. Ti_3_C_2_T_x_ solution with different concentrations was obtained by dissolving Ti_3_C_2_T_x_ in deionized (DI) water. The detail process of Ti_3_C_2_T_x_ synthesis was described in the supporting information.

To synthesis Ti_3_C_2_T_x_/PmPD, 1 g mPD monomer was firstly dissolved in 30 mL DI water, and added to a certain concentration of 100 mL Ti_3_C_2_T_x_ dispersion. The above mixture was continuously sonicated and stirred for 30 minutes. After that, Na_2_S_2_O_8_ solution (20 mL, 0.11 g mL^−1^) was slowly added to the above solution, and the reaction was maintained for 4 h, at −4 °C by ice bath. Ti_3_C_2_T_x_/PmPD composites with different mass ratios of mPD to Ti_3_C_2_T_x_ were prepared in turn by changing the concentration of Ti_3_C_2_T_x_. The obtained composites were labeled Ti_3_C_2_T_x_/PmPD-X (mass ratios X = 2/1, 5/1, 10/1). The final sediment was centrifuged, rinsed with amount DI water, and dried under vacuum (−55 °C, 12 h). Finally, Ti_3_C_2_T_x_/PmPD-X composites were obtained.

### 2.3. Characterization

The morphology and structure of as-obtained composites were characterized by scanning electron microscope (SEM, FEI Nova NanoSEM 230, FEI company, Hillsboro, OR, USA), scanning transmission electron microscope (STEM-EDS, JEM-2100F, Japan Electronics Co., Ltd. (JEOL), Tokyo, Japan), atomic force microscope (AFM, NanoMan VS, Bruker, Germany), X-ray powder diffraction patterns (XRD, D/max 2550 VB + XX diffractometer, Rigaku International Corp, Tokyo, Japan), X-ray photoelectron spectroscopy (XPS, K-Alpha 1063, Thermo Scientific, Waltham, MA, USA), Raman scattering spectra (532 nm, Renishaw inVia, Renishaw, London, England), and Fourier transformed infrared spectra (FT-IR, Nicolet IS10, Thermo Scientific, Waltham, MA, USA). The contact angles were measured using a Date Physics JY-82C goniometer (Dingsheng testing machine testing equipment Co., Ltd, Jinan, China). Zeta potentials were recorded using a Malvern Nano-ZS Zetasizer. The N_2_ adsorption-deposition isotherms were measured by bjbuilder KUBO-X1000 (Beijing Builder Electronic Technology Co., Ltd., Beijing, China). 

### 2.4. Batch Experiments

Potassium dichromate (K_2_Cr_2_O_7_) was dissolved in DI water to obtain aqueous solutions with different Cr (VI) concentrations. The obtained composites (10 mg) were put into 100 mL polyethylene bottle with 20 mL Cr(VI) solution, then the mixture was shaken at 30 °C for 12 h at 180 rpm speed. UV−vis spectrophotometer (540 nm) was utilized to detect the residual Cr(VI) concentration. All the experimental data were the average values of three measurements, whose relative error was less than 5%.

## 3. Results and Discussion

### 3.1. Material Characterization

**Figure 1:**
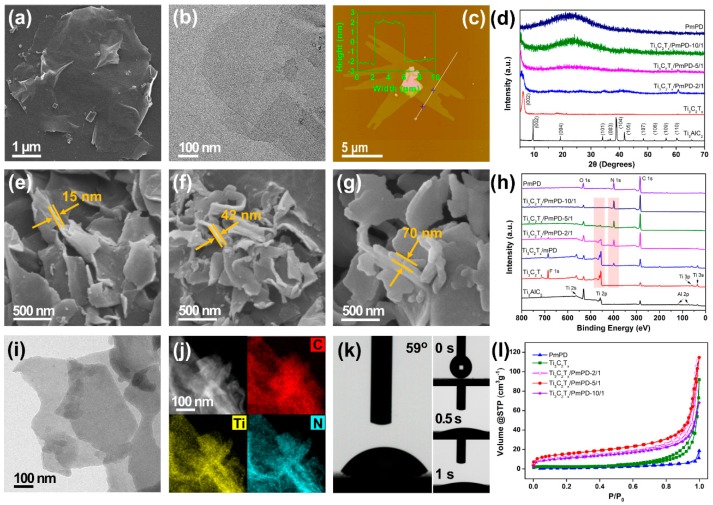
SEM images of (**a**) Ti_3_C_2_T_x_, (**e**) Ti_3_C_2_T_x_/PmPD-2/1, (**f**) Ti_3_C_2_T_x_/PmPD-5/1 and (**g**) Ti_3_C_2_T_x_/PmPD-10/1; TEM images of (**b**) Ti_3_C_2_T_x_ and (**i**) Ti_3_C_2_T_x_/PmPD-5/1; (**c**) AFM image of Ti_3_C_2_T_x_; (**d**) XRD patterns of Ti_3_AlC_2_, Ti_3_C_2_T_x_, Ti_3_C_2_T_x_/PmPD-X and PmPD; (**h**) XPS survey of Ti_3_AlC_2_, Ti_3_C_2_T_x_, PmPD, Ti_3_C_2_T_x_/mPD and Ti_3_C_2_T_x_/PmPD-X; (**j**) STEM-EDS mapping of Ti_3_C_2_T_x_/PmPD-5/1; (**k**) Water contact angle measurements of Ti_3_C_2_T_x_ (right) and Ti_3_C_2_T_x_/PmPD-5/1 (left). (**l**) N_2_ adsorption−desorption isotherms of Ti_3_C_2_T_x_, PmPD and Ti_3_C_2_T_x_/PmPD-X.

The structure and morphology of as-obtained composites were studied by using TEM, SEM, XRD, AFM, and XPS technologies. As can be seen from Figure 1a–c, Ti_3_C_2_T_x_ MXene exhibited 2D ultrathin morphology with a small average thickness of ~4 nm. The disappearance of the peak at 39° and the shift of the (002) peak to 6.04° also indicated the formation of Ti_3_C_2_T_x_ nanosheets (Figure 1d) [35,36]. As shown in Figure 1e–g, Ti_3_C_2_T_x_/PmPD-X composites showed a 2D dispersed and wrinkled morphology with a thickness ranging from ~15 nm to ~70 nm. In addition, taking Ti_3_C_2_T_x_/PmPD-5/1 as an example, Ti_3_C_2_T_x_/PmPD-X composites owned a thin and uniform shape (Figure 1i). The homogeneous dispersion of C, Ti, and N elements also revealed the homogeneous polymerization of PmPD on surface Ti_3_C_2_T_x_ nanosheets (Figure 1j). 

As can be seen from Figure 1d, the (002) peak of Ti_3_C_2_T_x_/PmPD-2/1 shifted to a smaller 2*θ* angle (5.54°) compared to that of Ti_3_C_2_T_x_ (6.04°). The decreased 2*θ* angle indicated a significant expansion of the interlayer spacing of Ti_3_C_2_T_x_/PmPD-2/1 from 14.6 to 15.9 Å. Furthermore, the (002) peak of Ti_3_C_2_T_x_/PmPD-5/1 shifted to 5.02° (17.6 Å interlayer spacing), which increased about 3.0 Å compared to that of original Ti_3_C_2_T_x_. In addition, with the further increase of mass ratio of mPD to Ti_3_C_2_T_x_, the (002) peak of Ti_3_C_2_T_x_/PmPD-10/1 would shift to the minimum 2*θ* angle, revealing that the interlayer spacing of Ti_3_C_2_T_x_ nanosheets were further expanded. The enlargement of interlayer spacing may be ascribed to the intercalation of PmPD in the polymerization process as well as the barrier effect of PmPD layer. These results revealed that the interlayer spacing of PmPD/Ti_3_C_2_T_x_ composites could be regulated from 14.6 to 17.6 Å, or even greater, by adjusting the mass ratio. It was noteworthy that the expansion of interlayer spacing would facilitate exposing the active sites and developing the adsorption potential of Ti_3_C_2_T_x_ MXene.

XPS spectrum were shown in Figure 1h. The presence of N element on intermediate Ti_3_C_2_T_x_/mPD indicated that mPD monomers were enriched on the Ti_3_C_2_T_x_ surface through electrostatic interaction and hydrogen bonding [37]. The enrichment of mPD was beneficial to the uniform polymerization of PmPD. The peak of N element was gradually enhanced with the increase of mass ratio of mPD to Ti_3_C_2_T_x_, which corresponded to the increase of composites thickness. In contrast, pure PmPD was homogeneously polymerized in solution and formed spherical shape with a diameter of 200–2000 nm (Figure S1). In contrast, no spherical morphology was observed in Ti_3_C_2_T_x_/PmPD composites, which further indicated the uniform polymerization of mPD on Ti_3_C_2_T_x_ surface.

In the contact angle experiments (Figure 1k right), the water droplet was immediately absorbed by Ti_3_C_2_T_x_ within ~1 s, which indicated that Ti_3_C_2_T_x_ had high hydrophilicity. It is commonly known that high hydrophilicity of Ti_3_C_2_T_x_ provides a good dispersion, which makes the adsorbent difficult to separate after treating pollutants [38]. In contrast, the contact angle of Ti_3_C_2_T_x_/PmPD increased to ~59° (Figure 1k left), which meant that the hydrophobicity of Ti_3_C_2_T_x_/PmPD was improved. The improvement of hydrophobicity was helpful to enhance the separation and recycling ability of Ti_3_C_2_T_x_/PmPD. N_2_ adsorption-desorption isotherms and calculated parameters were displayed in Figure 1l and Table 1, respectively. The specific surface areas of Ti_3_C_2_T_x_ and PmPD were 10.42 and 2.44 m^2^ g^−1^, respectively. The unfavorable specific surface area was probably caused by serious restacking [30]. Nevertheless, the specific surface areas of Ti_3_C_2_T_x_/PmPD-X were far beyond that of Ti_3_C_2_T_x_ and PmPD. In addition, the specific surface area of Ti_3_C_2_T_x_/PmPD-5/1 was five and 23 times that of Ti_3_C_2_T_x_ and PmPD, respectively. The improvement of specific surface area may be due to the expansion of Ti_3_C_2_T_x_ interlayer spacing and the inhibition of the stacking degree, which was consistent with the XRD results.

**Figure 2:**
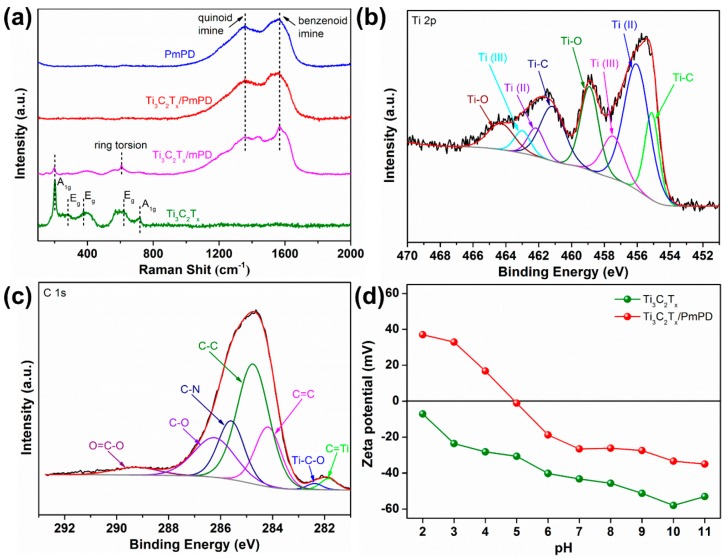
(**a**) Raman spectra of Ti_3_C_2_T_x_, PmPD, and Ti_3_C_2_T_x_/PmPD. High-resolution spectra of Ti 2p (**b**) and C 1s (**c**). (**d**) Zeta potentials of Ti_3_C_2_T_x_/PmPD and Ti_3_C_2_T_x_.

Raman spectra were recorded to further study the structure characteristics of as-obtained composites. As can be seen from Figure 2a, the modes of Ti_3_C_2_T_x_ at 200 and 718 cm^−1^ belonged to the vibrations of Ti and C, respectively. Moreover, the modes at 283, 375, and 618 cm^−1^ belonged to the vibrations of Ti [39]. With the enrichment of mPD, the main peaks of PmPD at 607, 1356 (quinoid imine), and 1567 cm^−1^ (benzenoid amine) appeared on intermediate Ti_3_C_2_T_x_/mPD [40]. After in situ polymerization of PmPD, the Ti_3_C_2_T_x_ peaks of Ti_3_C_2_T_x_/PmPD absolutely disappeared. The Raman spectrogram of Ti_3_C_2_T_x_/PmPD had two strong peaks similar to that of PmPD at ~1355 and ~1558 cm^−1^ [41], indicating the strong interaction between PmPD and Ti_3_C_2_T_x_.

The chemical composition of Ti_3_C_2_T_x_/PmPD were further investigated by XPS technology, and the high-resolution spectra of Ti 2p and C 1s are shown in Figure 2b,c, respectively. Furthermore, the peaks of Ti 2p_3/2_ at 455.0, 456.2, 457.5, and 458.8 eV originated from Ti-C, Ti(II), Ti(III), and Ti-O bonds, respectively. The peaks of Ti 2p_1/2_ at 461.2, 462.2, 463.0, and 464.3 eV were attributed to Ti-C, Ti(II), Ti(III), and Ti-O bonds, respectively [39,42]. The appearance of Ti(III) revealed that Ti_3_C_2_T_x_ was partially oxidized in the process of polymerization. The seven components centered of C 1s core level at 281.8, 282.4, 284.2, 284.8, 285.6, 286.3, and 289.3 eV were attributed to C-Ti, Ti-C-O, C=C, C-C, C-N, C-O, as well as O=C-O bonds, respectively [43,44].

Zeta potentials were recorded to study the surface charge property of Ti_3_C_2_T_x_ before and after functionalization. As seen in Figure 2d, Ti_3_C_2_T_x_ had a negative surface charge at a wide range of pH because of its terminating functional groups. However, the Ti_3_C_2_T_x_/PmPD showed a strongly positive surface charge when the pH value was less than 5. The strongly positive surface probably originated from protonated amino groups (-N^+^=) formed by attracting H^+^. Therefore, the conversion of Ti_3_C_2_T_x_ surface charge from negative to positive verified the successful modification. Ti_3_C_2_T_x_/PmPD with positive surface charge would show improved potential in removing anionic Cr(VI).

**Figure 3:**
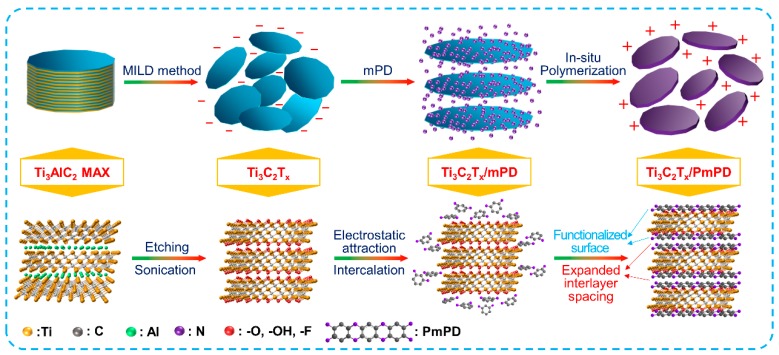
Preparation mechanism of Ti_3_C_2_T_x_/PmPD.

The preparation mechanism of Ti_3_C_2_T_x_/PmPD is illustrated in Figure 3. Firstly, Ti_3_C_2_T_x_ nanosheets were prepared from Ti_3_AlC_2_ MAX through the MILD method. In the process of functionalization, mPD monomers were attracted to the surface and interlayers of Ti_3_C_2_T_x_ nanosheets by electrostatic interaction and hydrogen bonding [37], These interactions induced the formation of intermediate Ti_3_C_2_T_x_/PmPD, namely Ti_3_C_2_T_x_/mPD. After that, mPD monomers were gradually polymerized on the surface and interlayers of Ti_3_C_2_T_x_ when adding oxidant, where Ti_3_C_2_T_x_ nanosheets served as templates or substrates. Finally, Ti_3_C_2_T_x_/PmPD composite with a positive surface charge and expanded interlayer spacing was obtained.

### 3.2. Adsorption Experiments

Adsorption performance of as-obtained composites was firstly investigated, as seen from Figure S2. The adsorption performance of Ti_3_C_2_T_x_/PmPD-X was higher than that of PmPD and Ti_3_C_2_T_x_. Moreover, Ti_3_C_2_T_x_/PmPD-5/1 owned the maximum removal capacity. The remarkable advantage of Ti_3_C_2_T_x_/PmPD-X probably originated to the synergistic effects. Therefore, Ti_3_C_2_T_x_/PmPD-5/1 was chosen as the representative of Ti_3_C_2_T_x_/PmPD in the following experiments. 

#### 3.2.1. Effect of pH 

**Figure 4:**
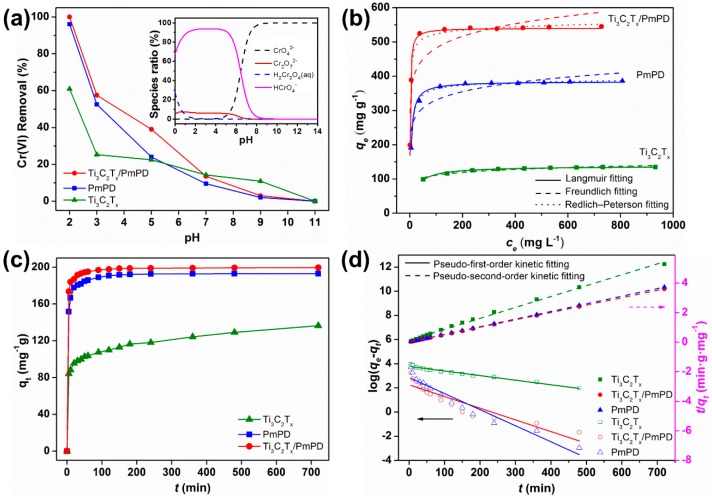
(**a**) Effects of pH (insets, the speciation diagram of Cr(VI) simulated by Visual MINTEQ); (**b**) Isotherms adsorption fitting; (**c**) Effect of adsorption time; (**d**) Pseudo-first-order kinetic model and pseudo-second-order kinetic model fitting.

The effect of pH on the removal performance of composites was investigated, as shown in Figure 4a. When decreasing pH value, the removal efficiency of Cr(VI) showed an upward trend. pH = 2 was the optimal condition, when Cr(VI) ions mainly existed in the forms of HCrO_4_^−^ (93.03 %) and Cr_2_O_7_^2−^ (6.42%) (Figure 4a inset) [45]. Low pH facilitated the formation of a strongly positive surface charge, and thus further enhanced the removal capacity of composites. In the next experiments, the optimal pH value was set to 2.

#### 3.2.2. Adsorption Isotherms

The adsorption isotherms were systematically investigated at different initial Cr(VI) concentrations. As seen in Figure 4b, with the increase of initial concentration, the adsorption capacity of PmPD, Ti_3_C_2_T_x_ and Ti_3_C_2_T_x_/PmPD increased, and finally reached saturation. The isotherms data were fitted by Langmuir, Freundlich, and Redlich–Peterson models to investigate the adsorption process and potential [46,47].

Figure 4b and Table S1 showed the isotherm parameters of the fitting models. The fitting coefficient of Redlich−Peterson model was higher than that of Langmuir and Freundlich model. Therefore, the adsorption behavior of Cr(VI) on Ti_3_C_2_T_x_/PmPD was appropriately simulated by the Redlich−Peterson model, indicating the hybrid adsorption process. The maximum theoretical adsorption capacity of Ti_3_C_2_T_x_/PmPD reached 540.47 mg g^−1^, exceed that of Ti_3_C_2_T_x_ (137.45 mg g^−1^) and pure PmPD (384.73 mg g^−1^). The excellent adsorption capacity of Ti_3_C_2_T_x_/PmPD also overstepped that of the reported MXene-based composites and other typical adsorbents, as can be seen from Table 2, indicating the brilliant application prospects of Ti_3_C_2_T_x_/PmPD.

#### 3.2.3. Adsorption Kinetics

The effects of contact time on the removal performance of composites were also studied, and the initial Cr(VI) concentration was 100 mg L^−1^. As seen from Figure 4c, Ti_3_C_2_T_x_/PmPD exhibited favorable removal performance than PmPD and Ti_3_C_2_T_x_. The removal efficiency of Ti_3_C_2_T_x_/PmPD reached 90% within 10 minutes. Moreover, the final removal efficiency was close to 100% within 120 minutes, thereby indicating the excellent adsorption rate. The kinetic data of Ti_3_C_2_T_x_/PmPD, PmPD, and Ti_3_C_2_T_x_ were fitted by pseudo-first-order and pseudo-second-order adsorption models [50,53]. According to the fitting results (Figure 4d and Table S2), the pseudo-second-order model was suitable for describing the adsorption process, indicating that the adsorption process was mainly involved chemical adsorption.

#### 3.2.4. Adsorption Mechanism

**Figure 5:**
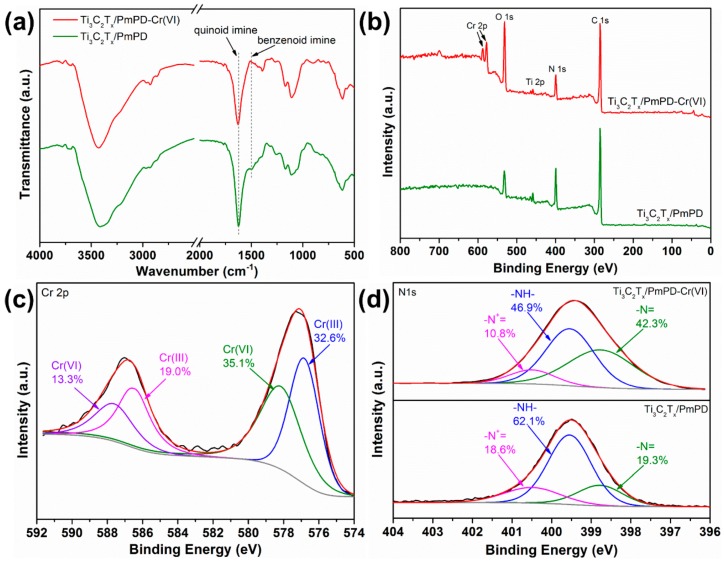
(**a**) FT-IR and (**b**) XPS survey spectra of Ti_3_C_2_T_x_/PmPD-Cr(VI) and Ti_3_C_2_T_x_/PmPD, respectively. XPS high-resolution of (**c**) Cr2p and (**d**) N1s.

To better understand the improved removal performance of Ti_3_C_2_T_x_/PmPD, the adsorption mechanism was investigated in detail. FT-IR spectra of Ti_3_C_2_T_x_/PmPD before and after treating Cr(VI) is shown in Figure 5a. The benzenoid amine peak (~1508 cm^−1^) of Ti_3_C_2_T_x_/PmPD-Cr(VI) was obviously decreased, and the quinoid imine peak (~1620 cm^−1^) was relatively enhanced, which implied that the oxidation state of Ti_3_C_2_T_x_/PmPD was improved after treating Cr(VI) [54].

Furthermore, the chemical compositions of Ti_3_C_2_T_x_/PmPD-Cr(VI) and Ti_3_C_2_T_x_/PmPD were determined by XPS to further illustrate the adsorption mechanism. As seen from Figure 5b, there were two peaks of Cr2p on Ti_3_C_2_T_x_/PmPD-Cr(VI), and the high-resolution spectrum of Cr2p was displayed in Figure 5c. The contributions at ~587.7 and ~577.6 eV originated from Cr(VI), while the contributions at ~586.4 and ~576.6 eV originated from Cr(III) [55]. The appearance of large amounts of Cr(III) (~51.6%) suggested that there was a redox reaction between Cr(VI) and Ti_3_C_2_T_x_/PmPD. As seen from Figure 5d, N1s peak of Ti_3_C_2_T_x_/PmPD were split into protonated quinoid imine at ~400.49 eV (19.3%), benzenoid amine at ~399.55 eV (62.1%), and quinoid imine at ~398.77 eV (18.6%), respectively [56]. After the treatment of Cr(VI), the percentage of benzenoid amine of Ti_3_C_2_T_x_/PmPD-Cr(VI) deceased from 62.1% to 46.9%, and the percentage of the quinoid imine increased from 19.3% to 42.3%. The results implied that there was a conversion of oxidation state from benzenoid amine to quinoid imine resulted from the oxidation of Cr(VI). Moreover, -N^+^= also occurred by doping positive Cr(III). It was noted that large percentage of benzenoid amines still existed after the treatment of Cr(VI). Hence, in the next adsorption cycle, Cr (VI) would also be converted into Cr (III) by existing benzenoid amines.

**Figure 6:**
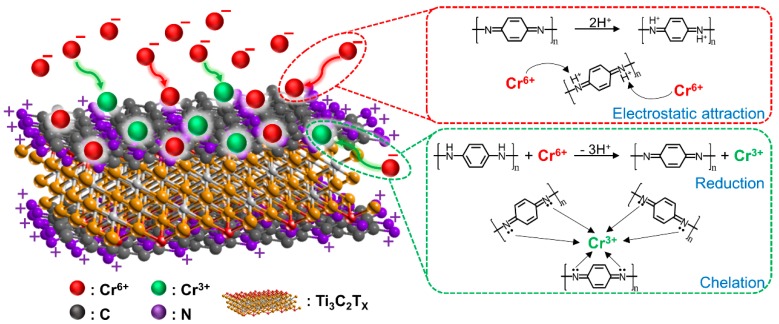
Cr (VI) adsorption mechanism of Ti_3_C_2_T_x_/PmPD.

Herein, the adsorption mechanism of Ti_3_C_2_T_x_/PmPD could be reasonably deduced, as shown in Figure 6. Firstly, anionic Cr(VI) was adsorbed onto Ti_3_C_2_T_x_/PmPD composite. Then, about 51.6% of Cr(VI) were converted to Cr(III) by benzenoid amine. At the same time, benzenoid amine was oxidized to quinoid imine by using Cr(VI). After that, Cr(III) was still adsorbed onto protonated quinoid imine of Ti_3_C_2_T_x_/PmPD composite through chelation. Hence, the adsorption process involved adsorption, reduction, and chelation interaction.

#### 3.2.5. Regeneration

**Figure 7:**
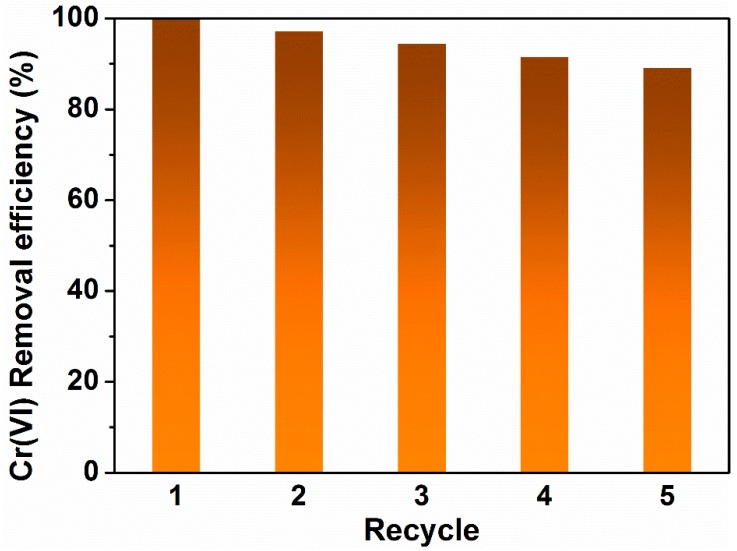
Regeneration of Ti_3_C_2_T_x_/PmPD.

The recycling ability of Ti_3_C_2_T_x_/PmPD was evaluated through adsorption-desorption experiments. After adsorption of Cr(VI), Ti_3_C_2_T_x_/PmPD was filtrated, rinsed with DI water, and then treated by NaOH solution (0.5 mol L^−1^) for the next cycle. As seen from Figure 7, the Cr(VI) removal efficiency still remained at ~90% after five recycle rounds with the initial Cr(VI) concentration of 100 ppm, revealing the favorable recycling performance of Ti_3_C_2_T_x_/PmPD.

## 4. Conclusions

This research developed a simple strategy to functionalize MXenes for efficient removal of Cr(VI). With the aid of PmPD, the surface charge of Ti_3_C_2_T_x_/PmPD was successfully converted from negative to positive. Furthermore, the interlayer spacing of Ti_3_C_2_T_x_/PmPD was enlarged from 14.6 to 17.6 Å, and the specific surface area of Ti_3_C_2_T_x_/PmPD was increased from 10.42 to 55.93 m^2^ g^−1^. These improvements indicated that the layer-by-layer restacking was successfully restrain. The maximum Cr(VI) adsorption of Ti_3_C_2_T_x_/PmPD was 540.47 mg g^−1^, which was superior to pure PmPD (384.73 mg g^−1^), Ti_3_C_2_T_x_ (137.45 mg g^−1^), and the reported MXene-based adsorbents. The excellent performance is attributed to the synergistic effects of Ti_3_C_2_T_x_ MXene and PmPD. The Cr(VI) adsorption mechanism mainly involved reduction, chelation, and electrostatic interaction. This study indicates that the strategy of in situ polymerization and intercalation was feasible and effective, which provides guidance for enhancing the performance of MXenes in the field of anionic pollutants. 

## Figures and Tables

**Table 1:** The specific surface area, pore volume, and average pore diameter parameters of Table 2. PmPD and Ti_3_C_2_T_x_.

Composites	S_BET_ (m^2^ g^−1^)	Pore Volume (cm^−3^ g^−1^)	Average Pore Diameter (nm)
Ti_3_C_2_T_x_	10.42	0.14	27.25
Ti_3_C_2_T_x_/PmPD-2/1	43.74	0.17	7.86
Ti_3_C_2_T_x_/PmPD-5/1	55.93	0.18	6.34
Ti_3_C_2_T_x_/PmPD-10/1	38.99	0.11	5.40
PmPD	2.44	0.029	23.54

**Table 2:** Comparison of removal performance of as-obtained Ti_3_C_2_T_x_/PmPD, MXene-based composites, and other typical adsorbents.

Adsorbents	Q_m_ (mg g^−1^)	pH	References
PDMDAAC	95.2	2	[48]
carbon nano-onions	23.5	3	[49]
Biochar	45.88	2	[50]
Fe@GA beads	33.9	3	[14]
nZVIRS700-Pd	117.1	3	[13]
Modified MXene	225	6	[51]
MXene	250	2	[52]
Ti_3_C_2_T_x_/PmPD	540.47	2	this work

## Supplementary Materials

The following are available online at https://www.mdpi.com/1660-4601/17/1/167/s1, Figure S1. SEM and TEM images of PmPD. Figure S2. Adsorption property of Ti_3_C_2_T_x_/PmPD-2/1, Ti_3_C_2_T_x_/PmPD-5/1, Ti_3_C_2_T_x_/PmPD-10/1, Ti_3_C_2_T_x_ and PmPD (initial Cr(VI) concentration 500 mg L^−1^ and 1000 mg L^−1^, pH = 2, temperature 30 °C). Table S1. Parameters of Langmuir, Freundlich and Redlich-Peterson isotherm models of Ti_3_C_2_T_x_/PmPD, Ti_3_C_2_T_x_, and PmPD. Table S2. Kinetic constants of the pseudo-second-order and pseudo-second order models of Cr(VI) on Ti_3_C_2_T_x_/PmPD, Ti_3_C_2_T_x_ and PmPD.
