# Supplementary material for: Two-Dimensional Titanium Carbides (Ti3C2Tx) Functionalized by Poly(m-phenylenediamine) for Efficient Adsorption and Reduction of Hexavalent Chromium
Source: Int J Environ Res Public Health. 2019 Dec 25;17(1):167. doi: 10.3390/ijerph17010167 (PMC6982338; doi:10.3390/ijerph17010167)
Supplement: Supplementary file 1 [file ijerph-17-00167-s001.pdf]

Two-dimensional titanium carbides ($Ti_3C_2T_x$) functionalized by poly(m-phenylenediamine) for efficient adsorption and reduction of hexavalent chromium

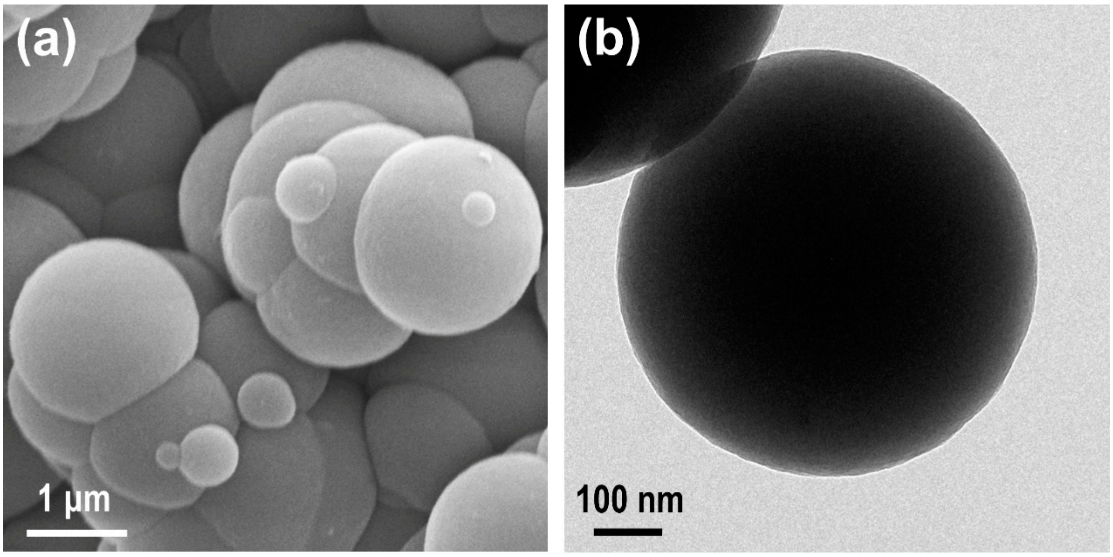

Figure S1. SEM and TEM images of PmPD.

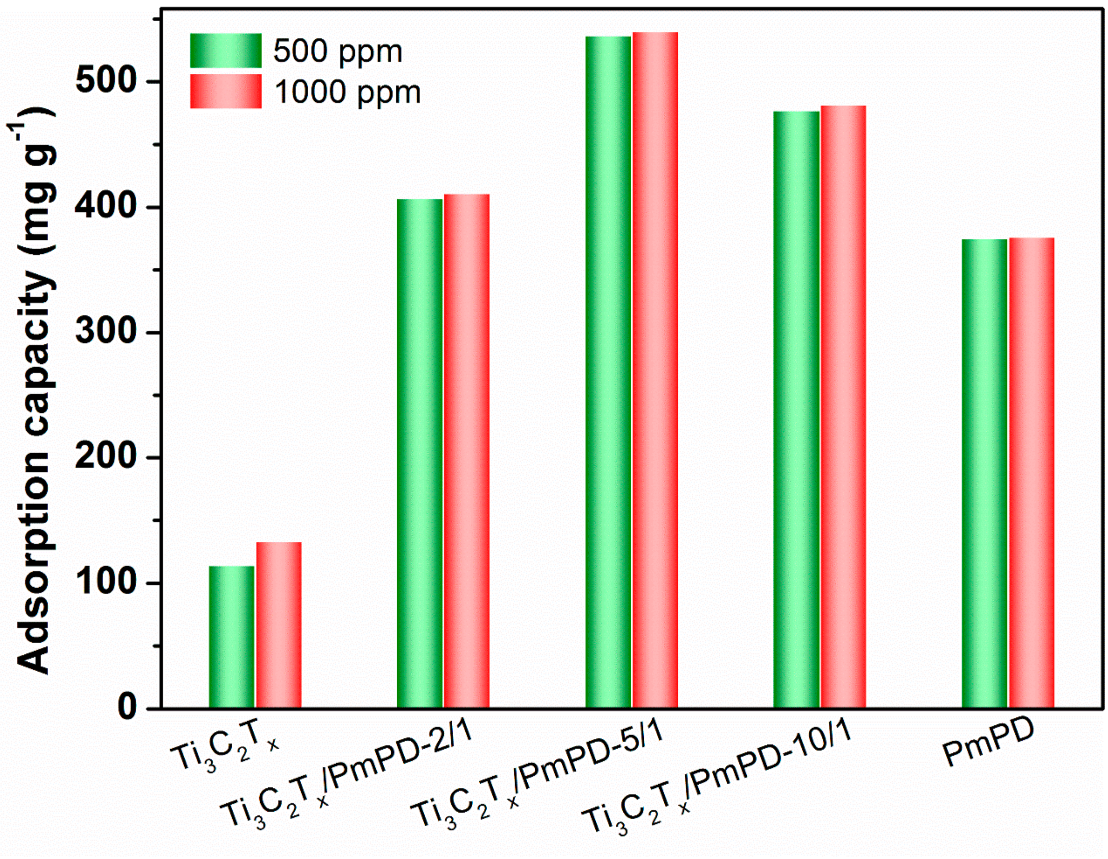

Figure S2. Adsorption property of $Ti_3C_2T_x$/PmPD-2/1, $Ti_3C_2T_x$/PmPD-5/1, $Ti_3C_2T_x$/PmPD-10/1, $Ti_3C_2T_x$ and PmPD (initial Cr(VI) concentration 500 mg L^{-1} and 1000 mg L^{-1}, pH = 2, temperature 30 ^{o}C).

Table 1. Parameters of Langmuir, Freundlich and Redlich-Peterson isotherm models of $Ti_3C_2T_x$/PmPD, $Ti_3C_2T_x$, and PmPD.

Composites	Langmuir			Freundlich			Redlich-Peterson			
	q_m	K_L	R^2	n	K_F	R^2	K	α	β	R^2
$Ti_3C_2T_x$/PmPD	540.47	0.7050	0.9731	10.1317	306.18	0.7766	474.00	0.9936	0.9776	0.9809
PmPD	384.73	0.2124	0.9931	9.7040	205.60	0.7888	89.84	0.2532	0.9863	0.9962
$Ti_3C_2T_x$	137.45	0.0471	0.9805	9.8068	69.47	0.9222	8.77	0.0787	0.9681	0.9918

Table S2. Kinetic constants of the pseudo-second-order and pseudo-second order models of Cr(VI) on $Ti_3C_2T_x$/PmPD, $Ti_3C_2T_x$ and PmPD.

Adsorbents	Pseudo-first-order model			Pseudo-second-order model		
	q_e	k_1	R^2	q_e	k_2	R^2
$Ti_3C_2T_x$/PmPD	9.55	0.0097	0.8537	200	$4.17*10^{-3}$	1
$Ti_3C_2T_x$	43.80	0.0038	0.9756	135.13	$4.04*10^{-4}$	0.9966
PmPD	17.06	0.0133	0.9402	192.31	$2.76*10^{-3}$	1
